# Mental Health Impacts of the COVID-19 Pandemic on College Students: A Literature Review with Emphasis on Vulnerable and Minority Populations

**DOI:** 10.3390/healthcare13131572

**Published:** 2025-06-30

**Authors:** Anna-Koralia Sakaretsanou, Maria Bakola, Taxiarchoula Chatzeli, Georgios Charalambous, Eleni Jelastopulu

**Affiliations:** 1 Department of Public Health, Medical School, University of Patras, 26504 Patras, Greece; 2 Master Programme in Health Management, Frederick University, Nicosia 1036, Cyprus

**Keywords:** COVID-19, mental health, university, student, minority

## Abstract

The COVID-19 pandemic significantly disrupted higher education worldwide, imposing strict isolation measures, transitioning learning online, and exacerbating existing social and economic inequalities. This literature review examines the pandemic’s impact on the mental health of college students, with a focus on those belonging to minority groups, including racial, ethnic, migrant, gender, sexuality-based, and low-income populations. While elevated levels of anxiety, depression, and loneliness were observed across all students, findings indicate that LGBTQ+ and low-income students faced the highest levels of psychological distress, due to compounded stressors such as family rejection, unsafe home environments, and financial insecurity. Racial and ethnic minority students reported increased experiences of discrimination and reduced access to culturally competent mental healthcare. International and migrant students were disproportionately affected by travel restrictions, legal uncertainties, and social disconnection. These disparities underscore the need for higher education institutions to implement targeted, inclusive mental health policies that account for the unique needs of at-risk student populations during health crises.

## 1. Introduction

Pandemics affect people’s lives in profound ways, particularly those of patients, as psychological resilience is tested. Physical and social isolation, disruption of daily routines, economic stress, food insecurity, and other stressors have been exacerbated by the COVID-19 pandemic, creating a situation in which individuals’ mental well-being and stability are likely to be threatened [1].

The uncertainty brought about by the pandemic has likely increased the frequency and severity of mental health issues globally [2]. Since the onset of the pandemic, many studies have already focused on studying its impacts on mental health [3,4]. International organizations have also highlighted the anticipated rise in mental health and substance use disorders during and after the pandemic as a critical public health concern. For instance, a national poll conducted by the American Psychological Association (APA) reported that over 36% of Americans believe the coronavirus has had a serious impact on their mental health [5].

Consequently, the COVID-19 pandemic has intensified interest in the psychological consequences of public health crises, prompting researchers and mental health professionals to focus on developing appropriate interventions and support strategies. This shift underscores the growing recognition of mental health as a fundamental component of overall healthcare during and after the pandemic.

To mitigate the spread of the virus, governments implemented various disease control measures, including school closures, social distancing, and home quarantine. These actions resulted in prolonged physical isolation for children and adolescents from peers, educators, extended family, and community networks. Quarantine among adults has also been associated with negative psychological effects such as confusion, anger, and post-traumatic stress. The duration of quarantine, fear of infection, lack of interest, frustration, lack of necessary supplies, lack of information, financial hardship, and social stigma appear to increase the risk of negative psychological effects.

In particular, children and adolescents, who are already at greater risk of developing mental health issues than adults, experienced additional stress related to health threats and financial instability. Social distancing and school closures were key contributors to increased loneliness among young people whose regular social interactions were restricted by public health measures. Loneliness—defined as the painful emotional experience resulting from the gap between actual and desired social connection—has emerged as a major concern. While social isolation is not necessarily synonymous with loneliness, early evidence in the context of COVID-19 suggests that more than one-third of adolescents reported high levels of loneliness, and nearly half of individuals aged 18–24 felt lonely during lockdown [6].

Several studies in the United States have examined student mental health during the COVID-19 pandemic, revealing a high prevalence of depression, stress, and anxiety. These effects have been especially pronounced among women, low-income students, and students from minority groups [7,8,9]. Numerous stressors have been identified as contributing factors, including concerns about personal and family health, difficulties concentrating, anxiety over academic performance, sleep disturbances, and diminished social interaction due to physical distancing [8].

During periods of social isolation, individuals are prone to experience increased levels of psychological distress [10]. Τwo of the most frequent mental health issues influencing millions of individuals globally every year are anxiety and depression, conditions frequently observed among undergraduate health science students. According to Agyapong-Opoku et al. (2023) [11], the prevalence of anxiety among undergraduate health science students ranges widely, with the lowest reported rate being 5.8% and the highest reaching 82.6%, yielding a median prevalence of 44.25%. Similarly, depression rates vary significantly, ranging from 2.1% to as high as 88.8%, with a median value of 34.8%. The relationship between anxiety and depression in these students is related to sociodemographic factors such as age, gender, personal relationships, ethnicity, and family history, as well as personal health conditions, academic, and socioeconomic issues [11].

Anxiety, depression, resilience, and other psychological factors appear to have affected students in China during the pandemic. A recent literature review has highlighted the prevalence of such issues, identified risk and protective factors, and analyzed the consequences for strategies aimed at improving mental health [12]. More specifically, students are experiencing these mental health issues at higher rates than before the pandemic began. Social isolation, irregular eating habits, work stress, the transition to online learning, and the uncertainty surrounding academic performance seemed to exacerbate their mental health struggles. Nonetheless, the use of new technologies and academic support systems appeared to foster psychological resilience.

Another study identified notable geographic differences in mental health outcomes. For example, non-Chinese college students exhibited higher rates of depression (60%) and anxiety (60%) compared to their Chinese counterparts, who reported 26% and 20%, respectively [13].

In addition to anxiety and depression, there was a notable rise in insomnia, obsessive–compulsive disorder, and suicidal ideation. According to another literature review, female students were more vulnerable to mental health disorders than male students. Although male students generally exhibited better stress management, they appeared to face a higher mortality risk associated with suicidal ideation [14].

Similar negative psychological effects were reported in studies involving international students. Zhao et al. (2022) [15] found that sleep issues, anxiety, and depression were prevalent (e.g., ~47%, ~40%, and ~49%, respectively, in a Korean sample), with other stress-related outcomes including loneliness, fear, and self-harm thoughts. These findings highlight the significantly elevated psychological distress among international students during the pandemic [15]. Additional risk factors such as living in rural areas, having a low socioeconomic status, and having relatives or friends in the healthcare sector were strongly correlated with adverse mental health outcomes [16].

Unfortunately, the findings demonstrate that academic student mental health is still in crisis even after the COVID-19 pandemic [17]. This ongoing situation underscores the urgent need for measures aimed at strengthening communication within academic institutions and fostering social connection among students [18]. To address these challenges, several recommendations have been proposed that may benefit the broader student population. First, universities should evaluate the effectiveness of mental health prevention and treatment programs across different age groups and between male and female students. Second, they should develop physical activity protocols that promote safe student participation. Third, administrators must proactively identify students at elevated risk for psychological distress—based on factors such as medical history—and collaborate with them to implement tailored support systems [19].

Although longitudinal data on the evolving impact of COVID-19 remains limited—particularly in light of vaccine availability and the emergence of new virus variants—mental health challenges among college students continue to be a critical area of concern. Given the pandemic’s dynamic nature, it is essential to monitor future trends and long-term psychological effects in order to design timely, adaptive, and effective interventions.

This literature review seeks to compile and analyze research data pertaining to college students who belong to national, ethnic, racial, migrant, gender, and sexuality-based minority groups, as well as those from low-income backgrounds. By doing so, it aims to shed light on the unique psychological burdens faced by these populations and identify contributing factors, in order to inform future preventive and intervention strategies.

While several studies have examined college student mental health in general, research specifically focusing on the impact of the COVID-19 pandemic remains relatively limited. This gap is even more pronounced when it comes to minority students, who are often underrepresented in the literature. In particular, there is a lack of quantitative research investigating the relationship between social isolation and psychological distress among college students from minority groups during the pandemic. This review aims to address this critical gap in the existing literature by centering on these underrepresented populations and analyzing the unique challenges they faced during this global crisis.

## 2. Materials and Methods

### 2.1. Literature Search

**Figure 1:**
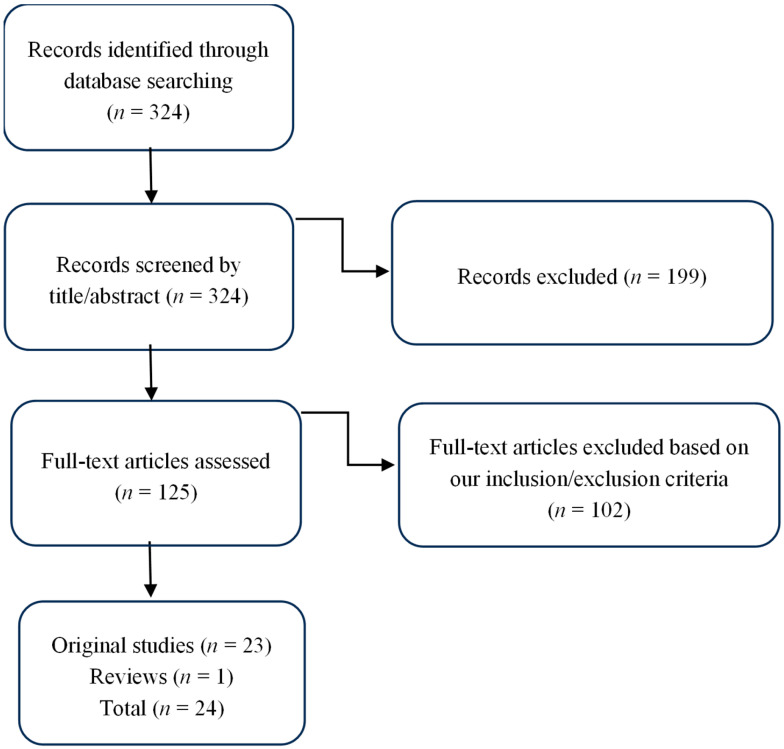
Flow diagram of study selection eligibility.

This literature review was conducted by searching databases including ScienceDirect, PubMed, and Google Scholar for relevant and original articles. Using the following keyword combinations, the search concentrated on English-written works: COVID-19, mental health, students, vulnerable groups, national minorities, racial minorities, migrant minorities, gender- and sexuality-based minorities, low-income students, LGBTQ+ youth, economic hardship, post-traumatic stress, anxiety, depression, and substance dependence. Articles were shortlisted for thorough reading depending on their relevance to the study’s main goals (Figure 1). The search was carried out at the beginning of November 2024 and included articles published over the preceding five years.

### 2.2. Inclusion and Exclusion Criteria

The inclusion criteria included the following: (a) publication in English; (b) full-text articles, including original studies and reviews; (c) focused on specified keywords; and (d) publication within the past five years. Exclusion criteria included the following: (a) non-English publications; (b) studies lacking data or available only as abstracts; (c) opinion articles or editorials; and (d) articles not directly related to the research topic or lacking clear conclusions.

## 3. Results

Prior to the detailed presentation of our findings, a summary table is provided to systematically delineate the key attributes of each included study (Table 1). This table encompasses Author, Country, Year, Population Size, Type of Article, and principal Outcomes relevant to the review aims, to facilitate a comprehensive understanding of the research scope and temporal distribution.

### 3.1. Mental Health of College Students Belonging to National, Ethnic, and Racial Minorities

Since the first year of the pandemic, minority students have experienced intense mental health challenges, such as depression and anxiety, with females and older students having higher chances of experiencing them [38].

Through the completion of an online questionnaire, an attempt was made to identify common mental health symptoms among Latin American, US Hispanic, and Spanish students, as well as clinical characteristics that predict higher post-traumatic stress symptoms (PTSS) in this population [30]. Before the pandemic, these levels of depression, suicidal ideation, and PTSS among college students were lower. Although familism was initially thought to be a protective factor, the study found that it could also function as a risk factor for PTSS, possibly due to increased caregiver responsibilities during the pandemic.

Another study showed that post-traumatic stress disorder was lower during the pandemic, while depression, alcohol use disorder, bulimia nervosa/binge-eating disorder, and comorbidity were higher [24]. More specifically, during the pandemic, students identifying as female were at especially high risk for elevated rates of alcohol use disorder, and students identifying as Black for major depressive disorder.

In North America, there were disproportionate effects on students of color, as Black and Latinx students were more likely to be personally affected by the pandemic, compared to White and Asian students. They reported the most consistent academic and financial impacts, expressing uncertainty about completing their current coursework and concerns about being able to afford food and return to college [26]. There were few differences between racial/ethnic groups in terms of changes in mental health symptoms, with all groups overall showing significant declines. The only notable difference was that Black students had lower average depression/anxiety scores compared to White students.

Another study investigated the pandemic’s impact on Arab higher-education students in Israel, examining social, academic, and financial factors [32]. Almost half of Arab students reported moderate to severe levels of depression (49.3%), anxiety (45.2%), and stress (54%). Moreover, women showed higher levels of depression and stress, job loss was a significant predictor of depression and anxiety, low income predicted depression, and COVID-19-related health concerns predicted anxiety. Additionally, poor online education, academic challenges, and pandemic-induced financial difficulties were all linked to increased stress, anxiety, and depression.

Both Arab and Asian students come from different cultural backgrounds and face difficulties with language, social differences, family alienation, and social integration into the new academic environment. A recent study involving international (44%), Asian (53%), Asian American (25%), and Hispanic/Latino and White/Caucasian (22%) students reported that a significant proportion of them experienced both anxiety and sadness daily. Furthermore, a strong positive correlation was found between overall well-being and mental health-related quality of life (r = 0.775, *p* < 0.001), confirming that higher levels of overall well-being are directly associated with improved mental health [37].

In a cross-sectional survey consisting of 16 items (both closed- and open-ended questions), 193 ethnically diverse college students participated [7]. Among students of color, 54% reported major disruptions in finances, 35% in living situations, 46% in academic performance, 49% in educational plans, and 36% in career goals. The main mental health issues reported were stress (41%), anxiety (33%), and depression (18%). Students also reported difficulties coping with racial injustice throughout the pandemic.

Additionally, Black students are traditionally less likely to seek mental health services than White students, which may have further increased their vulnerability during the pandemic. During the pandemic, social unrest and racial tensions continued to exist, further exacerbating mental health problems. One study reported that among Black students, 45.9% felt hopeless, 73.1% experienced exhaustion, 58.7% felt sadness, and 54.1% experienced overwhelming anxiety [27].

### 3.2. Mental Health of College Students Belonging to Migrant Minorities

Migrant students represent a distinct group because they often face multiple challenges, such as language barriers, cultural differences, limited social support, and difficulties accessing health and mental health services. Additionally, many of them have lower socioeconomic status and unstable or limited legal status in their country of study.

Resilience is a key factor in students’ mental health and their ability to adapt to challenging situations. Findings from a study show that the majority (92.23%) of migrant students at RAK Medical and Health Sciences University in the United Arab Emirates demonstrated moderate levels of resilience, which are considered normal. However, 6.31% exhibited low resilience, indicating that this group of students may be more vulnerable to stress and psychological pressure. Conversely, only 1.46% showed high resilience, suggesting that there is room for improvement through targeted interventions. These data confirm the importance of resilience as a protective mechanism for students’ mental health [39]. Nonetheless, there is still a lack of sufficient research specifically examining the mental well-being of migrant students during the COVID-19 pandemic and the unique challenges they faced throughout that period.

Enriquez et al. (2023) [34] examine whether and how self and parental immigration status contribute to Latina/o/x college students’ mental health and pandemic stressors. Quantitative data showed that the pandemic had widespread negative effects on mental health, regardless of students’ or their parents’ immigration status. Qualitative findings identified four main categories of pandemic stressors: financial insecurity, concerns about the COVID-19 virus, academic pressures, and social dynamics. Additionally, some undocumented students, along with their families, faced additional hardships due to exclusion from basic resources and public benefits, directly related to their lack of legal status [34].

Students specifically reported that federal pandemic relief excluded undocumented immigrants and their U.S.-citizen family members, denying them access to essential financial support. This exclusion, along with ineligibility for social safety nets (e.g., unemployment benefits, government food programs) and limited healthcare access, significantly hindered their ability to cope with pandemic-related financial and health stressors [34].

Ro et al. (2020) [25] conducted a study examining the impact of the COVID-19 pandemic on vulnerable student groups, such as migrants. Although the study has limitations, such as data being collected during the early phase of the pandemic, its large sample size provides valuable insights into the mental and physical health outcomes experienced by these populations. The findings suggest that migration status influenced health outcomes in complex ways. Contrary to expectations, undocumented students did not report worse health than citizens with migrant parents, while citizens with undocumented parents reported fewer health issues, possibly reflecting resilience. Furthermore, pre-pandemic use of university resources was associated with increased psychological and physical distress during the pandemic, whereas a sense of belonging generally correlated with better health outcomes, except among undocumented students, where this association was weaker [25].

### 3.3. Mental Health of College Students Belonging to Gender- and Sexuality-Based Minorities

During the COVID-19 pandemic, sexual and gender minority (SGM) youth were often forced to return to non-accepting family environments or faced limited access to healthcare, which significantly exacerbated their psychological distress. These students reported higher levels of psychological distress and poorer mental health outcomes compared to their cis-heterosexual peers. This aligns with existing research that documents elevated symptoms of depression, anxiety, and suicidal ideation among SGM populations. Additionally, individuals within the SGM community who experienced or witnessed discrimination or hostility based on race or ethnicity reported even worse mental health outcomes [33].

It was also observed that overall violence committed during the pandemic by either strangers or acquaintances against students decreased, which was not the case with sexual assaults by former or current partners, which remained unchanged. Notably, SGM students experienced increased sexual violence, which further negatively impacted their mental health [35].

Female students and people from sexual and gender minorities (such as LGBTQ+ students) faced particular difficulties, resulting in poorer mental health compared to the general student population. Based on studies, it appeared that gender plays an important role in how stress affects mental health, as women experienced higher levels of anxiety, depression, stress, and loneliness compared to male students [29].

LGBTQ+ students who have come out on campus may not follow the same tactics at home or in other social settings if they are hostile or unsupportive of their identity. During the pandemic, many of these students returned to such environments, leading to a worsening of mental health symptoms [20,21].

According to a survey, among LGBTQ+ students, 50.8% expressed feelings of hopelessness, 71.4% reported experiencing exhaustion, 65.1% mentioned feeling sad, and 52.4% stated that they experienced both overwhelming anxiety and anger [27]. Interestingly, while these levels are high, anxiety among LGBTQ+ students remained nearly unchanged from pre-pandemic levels, decreasing only marginally by 0.1%. Despite the elevated distress, LGBTQ+ students tended to hold more favorable views regarding the public health measures implemented to mitigate the pandemic’s effects, as reported in another study [40].

Turpin et al. (2023) [31] examined the syndemic phenomenon—the co-occurrence and interaction of multiple health and social problems that collectively intensify individual distress. Their study identified a syndemic comprising victimization, internalized LGBTQ+ stigma, racism, racialized heterosexism/cisgenderism, family rejection, and isolation, all of which were associated with increased psychological distress among SGM college students. The worsening of these syndemic factors since the start of the pandemic was strongly correlated with more severe mental health challenges [31].

A recent study examined how the COVID-19 pandemic impacted experiences of sexual and relationship violence among LGBTQ+ individuals in a college setting, as well as the prevention and support efforts aimed at this community. Through semi-structured virtual interviews with 31 professionals working in violence prevention, challenges such as social isolation, lack of privacy, unsafe living environments, and the marginalization of specific subgroups (e.g., queer and trans people of color) were highlighted. The shift to remote services created new difficulties but also opportunities for access to prevention programs. The findings emphasize the need for ongoing, tailored policies and practices to support LGBTQ+ individuals, even after the pandemic emergency has ended [41].

### 3.4. Mental Health of College Students from Low-Income Backgrounds

Students today experience increased difficulties, such as lack of housing and food, financial problems, isolation, feelings of non-acceptance, uncertainty about the future, and difficulties in accessing resources, which negatively affect their academic performance and mental health [21].

There appears to be a direct link between low or very low food security and housing insecurity, with an increased likelihood of moderate to severe symptoms of depression and generalized anxiety among students. For example, students experiencing food insecurity reported significantly higher rates of anxiety (75%) and depression (56%) compared to their food-secure peers (39.3% anxiety and 26.6% depression). The pandemic has exacerbated this situation, making mental disorders and lack of basic needs a serious and widespread problem [36].

Regarding the financial issue, before the pandemic, many students were employed, often in university-related positions, while a significant number maintained financial independence. The pandemic and the closure of university facilities led to massive job losses, causing severe financial hardship. As a result, many students faced increased difficulties in meeting basic needs, such as housing and food [21].

Financial pressure emerged as a central theme throughout the interviews, with half of the participants (50%) reporting financial stress. Specifically, 40% experienced anxiety and stress related to their finances, while 20% faced challenges related to employment during the pandemic; and 10% of participants reported experiencing both. These financial difficulties were linked to uncertainty, loss of income, and disrupted career plans, which collectively heightened stress during an already difficult period. For example, one privately funded international student delayed their study abroad process due to the lockdown. These findings illustrate the profound economic uncertainty affecting students’ mental health, emphasizing the need for targeted financial and psychological support for vulnerable student populations during crises [42].

The rising cost of tuition and living expenses, combined with student debt, has added to financial pressures, further impacting mental health and academic success. Many students must take on part-time work, making it challenging to balance employment and academic responsibilities, which in turn heightens stress levels. During crises such as the COVID-19 pandemic, this situation was worsened by a general lack of savings and often inadequate financial literacy [16].

Students from economically disadvantaged backgrounds faced disproportionate difficulties during the transition to distance learning. This format required high levels of self-discipline and autonomy, which widened learning gaps among students from varying socioeconomic backgrounds. Many struggled with limited access to technology, inadequate home learning environments, and intense psychological pressure. Concerns about maintaining academic performance and securing scholarships or financial aid only deepened their anxiety. Additionally, uncertainty about future employment opportunities, coupled with the broader instability caused by the pandemic, significantly affected these students’ mental well-being [28].

The study of Rudenstine et al. (2021) [22] assessed depression and anxiety symptoms among low-income public university students during the COVID-19 pandemic in New York City. It found high prevalence rates, with 63.1% experiencing severe depression and 52.2% severe anxiety among those exposed to high-level COVID-19-related stressors. Increased exposure to these stressors significantly predicted higher symptoms of both conditions. Additionally, having household savings of less than USD 5000 was linked to a higher risk of anxiety symptoms, highlighting the mental health impact on this vulnerable population [22].

Finally, research shows that despite the high prevalence of mental health problems related to financial stress, many students do not seek professional support. This limited engagement with mental health services may be attributed to social stigma, lack of awareness about available resources, and a tendency among students to cope with problems independently [23].

### 3.5. Diverging Patterns and Exceptions

Although the majority of studies reviewed consistently indicate heightened psychological distress among college students, especially those from minority or low-income backgrounds, some findings diverge from this general trend. For instance, Black students in certain studies reported lower average levels of depression and anxiety compared to their White peers, possibly due to differences in coping mechanisms or social support networks [24,26,27]. LGBTQ+ students, while overall showing elevated distress, did not exhibit a marked increase in anxiety compared to pre-pandemic levels in some samples, suggesting resilience or stable baseline distress levels [20,21,31,40].

Moreover, findings regarding undocumented students were mixed [25,34]; in one study, they did not report worse mental health outcomes than other migrant groups, potentially reflecting complex protective factors such as community solidarity or adaptive behaviors.

Additionally, familism, a cultural value often assumed to be protective in Hispanic communities, was found in some cases to be associated with increased post-traumatic stress, likely due to intensified caregiving burdens during the pandemic [30].

These exceptions highlight the heterogeneity of student experiences and underline the importance of contextual, intersectional analyses when assessing the mental health impacts of global crises.

While not undermining the general trend of widespread psychological distress, such divergent findings offer valuable insight into subgroup-specific dynamics and potential protective or risk-modifying factors.

## 4. Discussion

The COVID-19 pandemic imposed strict isolation measures, leading to the closure of educational institutions and the restriction of social contact. While this measure was deemed necessary to protect public health, it has also exacerbated existing social and economic inequalities.

For college students, lockdowns resulted in a significant reduction in face-to-face contact with peers and faculty, increasing feelings of loneliness and social isolation. These students also experienced increased levels of anxiety and stress, caused by the fear of infection, uncertainty about their future, and the pressures of maladjustment in the new educational environment.

At the same time, the feeling of being removed from the student community and the cancellation of important moments, such as graduation ceremonies, negatively affected students’ attachment to the university and their sense of belonging to a community [21].

The abrupt transition to digital learning further exposed disparities in access to technology and internet connectivity. Students from disadvantaged backgrounds faced considerable obstacles, intensifying educational inequality [21].

Between June and December 2020, a significant proportion of college students experienced mental health challenges, with 33% to 50% reporting at least one mental health condition and approximately 25% experiencing more than one. Nearly 50% reported worsened sleep, and 25% reported increased substance use. In the second wave, rates of depression and anxiety were 24.2% and 34.2%, respectively, higher than pre-pandemic national statistics of 21.5% and 27.6%. Similar increases were observed for eating disorders, obsessive–compulsive disorder, and post-traumatic stress disorder. Additionally, about one-third of students reported serious psychological distress, significantly higher than the 18% reported by students before the pandemic [38].

In light of these challenges, various coping strategies have been explored to mitigate psychological stress. Gaming can be a beneficial activity for college students, particularly during stressful times like the COVID-19 pandemic. Serving as an effective tool for stress relief, it can contribute positively to their overall mental health. According to a recent study, playing games significantly helps reduce psychological stress among college students during the pandemic, with analysis identifying gaming as one of the most effective mitigation measures, supported by statistical significance (*p* = 0.047). Given these findings, it would be valuable to extend this research to explore whether gaming could similarly benefit minority student populations, who may face additional stressors, to better understand its potential as an inclusive mental health support strategy [43].

Nonetheless, the impact of the pandemic has been uneven across student populations, disproportionately affecting those from marginalized and minority backgrounds. Already vulnerable due to systemic inequities, these individuals experienced heightened psychological distress during the pandemic. Discrimination, limited access to resources, and social exclusion intensified their mental health challenges, while stereotypes and bias further hindered their academic opportunities and prospects.

This review highlights the urgent need for targeted interventions to address the mental health needs of minority student populations. Educational institutions must prioritize mental health by promoting awareness, offering support services such as telecounseling, and creating inclusive environments that reduce stigma and encourage help-seeking behaviors [44].

In particular, public health emergency planners and university administrators should tailor strategies to support gender- and sexuality-based minorities, such as LGBTQ+ students, through personalized, inclusive mental health education and crisis response programs. Early and sensitive intervention is essential to safeguarding the psychosocial well-being of these students in future public health emergencies.

Finally, students from low-income backgrounds experienced disproportionately high levels of anxiety, depression, and stress due to the financial instability caused by the pandemic. Institutions must recognize the link between economic hardship and mental health, and respond with comprehensive support, both financial, through scholarships and basic needs assistance, and psychological, through accessible mental health services and telemedicine.

Some important factors were not adequately addressed in the studies reviewed. For instance, few investigations examined how COVID-19 vaccination affected students’ mental health either positively (e.g., reduction in anxiety) or negatively (e.g., vaccine hesitancy, social stigma) [40]. Similarly, the psychological impact of losing relatives or community members due to COVID-19-related deaths among minority students remains underexplored, despite the often disproportionately high mortality rates in these communities.

Despite the broad scope of the studies reviewed, several limitations must be acknowledged. First, there was significant heterogeneity among studies in terms of methodology, sample size, cultural context, and the timing of data collection during the pandemic. This diversity complicates the comparison of results and limits the generalizability of findings. Second, the majority of studies were cross-sectional, which prevents tracking the long-term evolution of students’ mental health and limits the ability to draw causal inferences. Third, although various minority student groups were examined, some subpopulations—such as students with disabilities (physical, intellectual, or mental) or individuals who do not identify within the gender binary—were underrepresented or entirely absent, highlighting the need for more inclusive and intersectional future research.

Additionally, most studies did not sufficiently differentiate students by academic level (undergraduate, graduate, doctoral), even though their needs, responsibilities, and sources of stress may differ significantly across these groups. These gaps emphasize the need for further targeted research that investigates the pandemic’s effects with greater stratification and depth.

## 5. Conclusions

The pandemic revealed and intensified existing inequalities within higher education, disproportionately affecting minority and economically disadvantaged students. As we look toward the future, it is essential that higher education systems become more resilient and equitable, ensuring that all students—regardless of background—can access support and succeed even in times of crisis.

To achieve this, future research should focus on identifying and evaluating effective, inclusive strategies and interventions that promote psychological resilience, support mental well-being, and foster equity within diverse student populations. This includes exploring tailored support, assessing long-term outcomes of programs, and developing innovative tools to monitor and enhance student well-being.

By strengthening the evidence base, institutions will be better equipped to prepare for and respond to future challenges, ensuring every student has the opportunity to thrive.

## Figures and Tables

**Table 1:** Summary table of studies included in the present review.

Author/Country/Year	Population Size/Type of Article	Outcomes and Findings Related to Review Aim
Campbell et al./USA/2020 [20]	USA college students (no specific population size reported)/ Research article	- The article does not specifically mention the effects of the COVID-19 pandemic on minority groups in relation to family violence. - However, it is widely recognized that minority communities often face heightened risks during crises, including increased vulnerability to domestic violence. - Strengthening community partnerships is key to supporting minority groups affected by family violence during the pandemic.
Lederer et al./USA/2020 [21]	USA college students (no specific population size reported)/ Research article	- Increased rates of housing and food insecurity, financial hardships, lack of social connectedness, and uncertainty about the future during the COVID-19 pandemic. - These issues may be exacerbated for students of color and low-income students, indicating a focus on the inequalities within this population.
Rudenstine et al./USA/2021 [22]	N = 1821 university students/ Quantitative study	- High prevalence of depression and anxiety symptoms during COVID-19. - High prevalence of PTSD symptoms; distress was associated with exposure to pandemic-related stressors. - Specific stressors like financial strain and social isolation significantly predicted symptom severity.
Lee et al./USA/2021 [23]	N = 1412 undergraduate students/ Quantitative study (online survey study)	- High prevalence of stress (88%), anxiety (44%), and depression (36%) among students during the early COVID-19 phase. - Female, rural, low-income, and academically underperforming students were more vulnerable.
Kim et al./USA/2021 [24]	N = 8613 college students (*n* = 3643 pre-pandemic, *n* = 4970 during pandemic)/Original quantitative study	- Increased rates of depression (OR 1.32), alcohol use disorder (OR 1.70), bulimia nervosa/binge-eating disorder (OR 1.54), and comorbidity (OR 1.19) during the pandemic compared to before. - Decreased rates of PTSD (OR 0.86) during the pandemic. - Women showed a stronger upward trend in alcohol use disorder (OR 1.47). - Black students showed a stronger upward trend in depression (OR 1.72). - No significant changes for anxiety disorders, insomnia, anorexia nervosa, or suicidality.
Ro et al./USA/2021 [25]	N = 2111 California college students (undergraduates)/Quantitative study	- Students with undocumented parents were least likely to report COVID-19-related mental/physical health effects. - No significant difference was found between undocumented students and those with lawfully present parents. - Greater campus resource use and higher sense of belonging were paradoxically linked to worse health outcomes. - Widespread negative health effects were observed, and loss of access to campus-based support during the early pandemic stages was harmful.
Molock and Parchem/USA/2021 [7]	N = 193 ethnically diverse college students/ Cross-sectional survey study	- Students of color reported significant disruptions: finances (54%), living situation (35%), academic performance (46%), educational plans (49%), career goals (36%). - Mental health challenges: stress (41%), anxiety (33%), depression (18%). - Difficulties managing racial injustice during COVID-19.
Reyes-Portillo et al./USA/2022 [26]	N = 4714 college students from 55 universities in New York and New Jersey/ Online survey conducted	- Majority female (76.1%), diverse racial/ethnic composition (44% White, 23% Latinx, 18% Asian, 8% Black, others). - During COVID-19, higher rates of depression, anxiety, and PTSD symptoms reported. - Minority students showed disparities in mental health outcomes. - Female students reported greater psychological distress. - Mental health severely affected by COVID-19 with disproportionate impact on students of color. - Key stressors: worry about infection, financial and academic challenges, loneliness.
Wood et al./USA/2022 [27]	N = 489 U.S. college students, undergraduates/ Cross-sectional online survey	- 81.6% reported at least one negative mental health symptom during COVID-19. - Increases in hopelessness (+7.8%), loneliness (+6.7%), sadness (+8.8%), depression (+2.6%), anxiety (+5.2%), anger (+14.6%). - LGBTQ and Black students experienced significantly more symptoms than straight and White students. - Highlights disproportionate mental health impact on marginalized groups during the pandemic.
Rodríguez-Planas/ USA/2022 [28]	N ≈ 12,000 academic records/ Research study (Quantitative methods: difference-in-differences models and event study analyses)	- An income differential effect of the COVID-19 pandemic on college students’ GPA, indicating that students from lower-income backgrounds were more adversely affected academically during the pandemic.
Buizza et al./Italy/2022 [29]	N = 20,108 college students/ Systematic review of 17 longitudinal studies	- Increased anxiety, mood disorders, and distress during COVID-19. - Higher alcohol use, sedentary behavior, and excessive internet use. - Decreased physical activity. - Female and SGM students showed worse mental health outcomes.
Torres et al./USA/ Mexico/ Chile/ Ecuador/ Spain/ 2023 [30]	N = 1113 college students (USA, Mexico, Chile, Ecuador, Spain)/ Cross-sectional, online survey study	- Focused on Latin American, US Hispanic, and Spanish students. - Found elevated depression, suicidality (29.4%), and post-traumatic stress symptoms compared to pre-pandemic norms; clinical anxiety levels were <5%. - Regression showed depression, loneliness, perceived stress, anxiety, and coping strategies explained 62% of PTSS variance; age, mental health history, social support, and familism did not predict PTSS. - Stresses need for culturally sensitive, cost-effective prevention/intervention strategies for racially/ethnically minoritized college students.
Turpin et al./ USA/2022 [31]	N = 565 sexual and gender minority (SGM) College students/Quantitative cross-sectional study with latent profile analysis	- Identified syndemic profiles with differing psychological distress levels, worsened during the pandemic. - Positive associations found between syndemic factors (family rejection, isolation, victimization, stigma, racism) and psychological distress. - Racism and isolation showed the strongest effects.
Alfayumi-Zeadna et al./Israel/2022 [32]	N = 420 Arab minority university students/ Cross-sectional quantitative study	- High rates of moderate to severe depression (49.3%), anxiety (45.2%), and stress (54%). - Mental health impacted by low online learning quality, academic and economic difficulties. - Women, job loss, low income, and COVID-19-related health concerns predicted worse mental health.
Lin et al./USA/2023 [33]	N = 139,470 U.S. college students across 60 campuses/ Quantitative survey with multivariable regression	- Sexual and gender minority (SGM) students reported higher depression, anxiety, and suicidal ideation than cisgender heterosexual peers. - Discrimination (e.g., racism) further worsened mental health in SGM individuals. - High social cohesion was protective for SGM (reduced odds of depression OR 0.59 and anxiety OR 0.69), but low cohesion increased risk in cis-hetero students (depression OR 1.37, anxiety OR 1.32).
Enriquez et al./USA/2023 [34]	Ν = 1600 Latina/o/x University of California Undergraduates/ mixed-methods (quantitative and qualitative)	- Quantitative: high levels of negative mental health impacts due to COVID-19 (mean 2.46–2.59 = “moderate to a lot”); no statistically significant differences between undocumented, citizen-with-undocumented-parent, and citizen-with-lawful-parent groups. - Qualitative: four main stressors identified: financial insecurity, virus-related fears, remote learning challenges, and social dynamics. - Legal vulnerability created unique experiences for undocumented students and those with undocumented parents. Immigration status influenced coping ability due to limited access to resources.
Deng et al./USA/2023 [35]	N ≈ 217,552 college Population (0.12% transgender women, 0.21% transgender men, and 1.97% gender-diverse individuals)/ Quantitative longitudinal study	- Patterns of violence and mental health outcomes showed increased vulnerability among SGM students before and during COVID-19. - Pandemic stressors and discrimination were linked to worsened mental health.
Soria/USA/2023 [36]	N = 49,122 college students from 130 community colleges and 72 four-year institutions/ Quantitative study	- Did not specifically focus on minorities or analyze data by race or ethnicity. However, the findings on increased depression and anxiety linked to basic needs insecurity likely apply to all students, including minorities.
Wei/USA/2024 [37]	N = 32 college students (53% Asian, 25% Asian American, 13% Hispanic/Latino, 9% White/Caucasian)/ Original quantitative study	- Mental health and well-being assessed via PERMA-Profiler and MHQoL scales. - Positive correlation between PERMA (well-being) and MHQoL (mental health) scores (r = 0.775, *p* < 0.001). - High scores in positive emotions, engagement, relationships, meaning, accomplishment, and health (PERMA components). - Co-existence of positive and negative emotions. - Connectedness to family and friends significantly related to PERMA scores, but not MHQoL. - No significant differences by ethnicity, age, or gender; Asian students had slightly higher mental health scores. - No strong evidence that Asian international students had worse mental health than others.
Hotez et al./USA/2022 [38]	N = 128 (June 2020), N = 240 (December 2020); undergraduate minority students/ Mixed methods (quantitative and qualitative)	- Majority non-White (66–70%) and female (71–73%). - Increase in depression, anxiety, social anxiety at wave 2 (3–4 times higher). - Females reported more anxiety; non-White students less substance use. - Main stressors: social isolation, academics, relationships, job insecurity, COVID-19 fears.
Salgado et al./ UAE/2024 [39]	N = 206 university students who migrated/ Empirical study	- 92.23% had normal resilience, 1.46% high resilience. - 84.95% had high positive mental health. - Significant positive correlation between mental health and resilience (r = 0.188, *p* = 0.007).
Xu et al./USA/2024 [40]	N = 611 university students (79% straight, 20% LGBTQ+)/ Quantitative web-based survey	- LGBTQ+ students exhibited higher levels of anxiety and fear compared to non-LGBTQ+ groups. - LGBTQ+ students were more negatively impacted by COVID-19, but had more positive views on public health measures (*p* = 0.001).
Klein et al./USA/ 2024 [41]	N = 31 LGBTQ+ campus antiviolence personnel/Qualitative study (semi-structured interviews)	- COVID-19 highlighted disparities in sexual and relationship violence (SRV) against LGBTQ+ students. - Pandemic increased social isolation, reduced privacy, and limited access to support.
Ma et al./JAPAN/ 2025 [42]	N = 20 international students/ Qualitative study	- International students faced multiple stressors during COVID-19 including financial pressure, xenophobia, and fear of infection.

## Data Availability

All data related to this study are provided in the manuscript. No new data were created or analyzed in this study.
